# Where are the non-sovereign islands in health policy and systems research?

**DOI:** 10.1136/bmjgh-2026-025304

**Published:** 2026-08-12

**Authors:** Robert A J Borst

**Affiliations:** 1 Erasmus School of Health Policy and Management, Erasmus University Rotterdam, Rotterdam, the Netherlands

**Keywords:** Health systems, Universal Health Care, Global Health

**Summary box**

- Global health policy and systems research largely ignores small non-sovereign islands, despite these islands facing significant and unique challenges.
- Existing frameworks generally exclude islands or compress them into a single narrative with metropolitan political economies.
- Health system challenges on non-sovereign islands stem from political relationships with metropolitan states, not smallness alone.
- Decolonising global health requires research that treats islandness as an analytic category by itself.
- A decolonial research agenda for non-sovereign islands must be rooted in island priorities.

The disability caused by the extraction of wealth and the subjugation of people bred a persistent poverty that has endured. Unless the fundamental obstacle (sic) to our development are addressed, I fear that that imbalance of power and wealth in the global community of nations will remain. – Mia Motley^1^

Over 10% of the world’s population lives on islands.^2^ Yet in global health policy and systems research (HPSR), islands remain overlooked peripheries. This holds especially true for small, often formerly colonised, partially independent and generally tropical islands or island archipelagoes.^3 4^ Many of these islands remain attached to their formerly colonising metropolitan state. Hence, such islands are often referred to as ‘subnational island jurisdictions’ or ‘non-sovereign islands’.^5^ The term ‘non-sovereign island’ is used in reference to a ‘mainland-island relation’^5^ whereby islands have some degree of self-government while a significant part of the decision-making power remains with a metropolitan polity. As I will argue in this commentary, it is precisely this governance arrangement that is causing unique problems to non-sovereign islands’ health systems as well as undermining possible solutions—thus demanding more dedicated HPSR.

The absence of island studies in HPSR is difficult to justify. In light of the impetus to morally and methodologically decolonise global HPSR scholarship and practice,^6^ this holds particularly true for non-sovereign islands that remain in a relationship with their former coloniser. Island jurisdictions in general suffer from what the Prime Minister of Barbados, the Honourable Mia Mottley, calls an enduring ‘disability’, including scientifically. Non-sovereign islands in particular are often denied access to the systems of knowledge production and utilisation that many metropolitan territories take for granted. For instance, a review of health research in Guam and the Cook Islands found most studies had international corresponding authors and only half included local co-authors,^7^ and a Dutch policy report shows that Dutch Caribbean universities receive significantly less research funding than their metropolitan counterparts.^8^ This highlights that a critical and reflexive engagement of HPSR with islands is long overdue.

Substantively, small islands present a distinctive set of health system challenges that have long been acknowledged^9^ but remain insufficiently studied. Geographic isolation, small populations, undiversified economies of scale and chronic workforce shortages are often accompanied by disproportionate exposure to the effects of climate change and geopolitical instability.^10^ The small islands of St Eustatius and Saba, for instance, rely on erratic supply chains and interrupted off-island medical referrals by helicopter or plane on a daily basis.^11^ While such challenges have been described before, there is little effort to design and implement workable policy responses to them. The assumption tends to be that health system interventions developed for mainland contexts will yield similar results on islands, although on a smaller scale. This assumption of scalability is applied to islands in general^12^ and is rarely examined by the researchers, donors and policymakers who make it.

The idea that islands are merely smaller geographies rather than distinctive sociocultural entities cannot be separated from enduring coloniality. The Spanish colonisers classified the Caribbean islands Aruba, Bonaire and Curaçao jointly as *las islas inútiles*: the useless islands.^13^ To this day, many small islands worldwide are approached through governance arrangements that reproduce this logic, treating islands as administratively inconvenient or economically peripheral. Health system stewardship is often fragmented across local and metropolitan levels: local health system actors’ fiscal autonomy is constrained by metropolitan state budgetary frameworks and national health policy priorities may be misaligned with epidemiological realities on the islands. These problems thus affect how islands’ health systems can plan, adapt and respond over time.

In much research, including global health, islands are often approached through the supranational Small Island Developing States (SIDS) frame. Since its formal recognition by the United Nations in the early 1990s, the SIDS category has provided a shared vocabulary for discussing vulnerabilities, economic constraints and international cooperation among small island geographies. In global health research, the SIDS framework has also facilitated the identification of common challenges in fields like non-communicable diseases, climate adaptation and human resources for health planning. However, while the framework does facilitate comparative HPSR analyses, it also reinforces the idea of islands as a coherent and universal group with comparable health system challenges. Comparing Puerto Rico to Nauru makes as much sense as comparing Luxembourg to Venezuela.

Analytically, the SIDS framework also remains quite shallow. Although strictly not a list of ‘states’, it risks reproducing the sovereign state ideal, thereby excluding islands such as St Barthélemy and Saint-Martin by design. Overseas territories, for instance, are rendered conceptually anomalous, despite facing significant constraints. The framework also compresses diverse political economies, governance arrangements and historical trajectories into a single story of fragility or ‘development’. In doing so, it sustains a reductionist narrative in which smallness is treated as an inherent characteristic rather than as an outcome of specific political and institutional arrangements.^14^ While the diplomatic utility of the SIDS framework, and in particular its capacity to produce ‘strength in unity’, should not be dismissed, its dominance in the literature has crowded out more fine-grained analyses of how health systems actually function in different island contexts.

What is needed is an HPSR research agenda built around the specific conditions of non-sovereign small islands, instead of frameworks designed in metropolitan states. Such an agenda would have three connected priorities. First, it would invest in situated, locally embedded research that takes islandness seriously as an analytic. Such research could, for instance, study how fiscal dependency on metropolitan states shapes investment in trends such as personalised medicine, or how healthcare regulation operates when decision-making authority is split across jurisdictions. Second, it would attend to what health systems on these islands produce, not to what they lack. Informal task-shifting, cross-institutional mobility and community-based resilience are features of daily health system functioning on many small islands.^9–11^ Understanding how and why they work is a precondition for any meaningful policy response. Besides, these could also offer inspiration to many of their metropolitan counterparts who are now increasingly confronted with shortages as well. Third, this agenda ought to study what it means to train and maintain a local health workforce, regardless of scale, rather than simply relying on training capacity in metropolitan states.

None of this is straightforward. Furthermore, a decolonial research agenda requires honesty about the risk of reproducing the very dynamics it seeks to unsettle. Research conducted on non-sovereign islands by visiting scholars affiliated with metropolitan institutions is a well-worn pattern that strengthens privileged knowledge systems.^15^ The alternative could be to build and nurture long-lasting reciprocal and dignified relations between researchers, policymakers and practitioners that span the divide between metropolitan states and ‘overseas’ territory. It also requires structuring research programmes so that the questions being asked are rooted in island priorities, developing analytical capacity within island institutions and translating knowledge to local policy processes. Concretely, this means funding long-term research partnerships between metropolitan and island universities, supporting island-based postgraduate training in HPSR and creating mechanisms for local health system actors to actively commission and use research—including embedded research. Journals and funders equally have a role, for instance, by facilitating young researchers from small island geographies with special issues and small travel grants for training, fieldwork and conferences, respectively.

If HPSR is serious about decolonising its empirical foundations, the worlds that sit between full sovereignty and colonisation can no longer be treated as residual categories. Small non-sovereign islands are not rare cases and should not be treated as the end in a knowledge transfer equation. Small non-sovereign islands are home to people who live within these unique but often liminal geographies of contested power and governance and who have the same right to health as people living in metropolitan states. This is precisely what makes small islands worth studying on their own terms. Enacting an HPSR research agenda that takes small islands seriously should be centre stage in global health’s endeavours to decolonise.

## Data Availability

All data relevant to the study are included in the article.
